# Typhoidal salmonella disease in Mukuru informal settlement, Nairobi Kenya; carriage, diversity, and antimicrobial resistant genes

**DOI:** 10.1371/journal.pone.0298635

**Published:** 2024-02-23

**Authors:** Purity Kasiano, Susan Kavai, Susan Kiiru, Andrew Nyerere, Samuel Kariuki

**Affiliations:** 1 Kenya Medical Research Institute, Centre for Microbiology Research, Nairobi, Kenya; 2 Jomo Kenyatta University of Agriculture and Technology, JKUAT, Nairobi, Kenya; Kafrelsheikh University Faculty of Veterinary Medicine, EGYPT

## Abstract

**Introduction:**

Multiple studies have shown that typhoid fever is endemic in developing countries characterized by poor hygiene. A unique way of *Salmonella* Typhi (*S*.Typhi) pathogenicity is establishing a persistent, usually asymptomatic carrier state in some infected individuals who excrete large numbers of bacteria in faeces. This study aimed to determine the isolation rate of *S*.Typhi from blood and stool samples among cases and asymptomatic individuals in the Mukuru informal settlement and identify antibiotic resistance patterns within the same population.

**Materials and methods:**

We recruited 1014 outpatient participants presenting with typhoid-like symptoms in selected health centres in Nairobi, Kenya. Bacterial isolation was done on Xylose Lysine Deoxycholate agar (XLD) and Mac Conkey agar (Oxoid), followed by standard biochemical tests. Identification was done using API20E, and *S*.Typhi was confirmed by serotyping using polyvalent antisera 0–9 and monovalent antisera d. The Kirby-Bauer disc diffusion method was used to test the antimicrobial susceptibility of *S*.Typhi isolates, while Multi-Drug Resistant (MDR) strains were characterized using conventional PCR.

**Results:**

Of 1014 participants, 54 (5%) tested positive for *S*.Typhi. Thirty-eight (70%) of the *S*.Typhi isolated were from stool samples, while sixteen (30%) were from blood. Three (0.2%) of the isolates were from asymptomatic carriers. Of the 54 *S*.Typhi isolates, 20 (37%) were MDR. Resistance to ciprofloxacin and nalidixic acid was 43% and 52%, respectively. Resistance to amoxicillin-clavulanic acid (a beta-lactam inhibitor) was 2%. The *Bla*_TEM-1_ gene was present in 19/20 (95%) MDR isolates.

**Conclusion:**

MDR *S*.Typhi is prevalent in Mukuru Informal settlement. The sharp increase in nalidixic acid resistance is an indication of reduced susceptibility to fluoroquinolones, which are currently the recommended drugs for the treatment of typhoid fever. This study highlights the need for effective antimicrobial stewardship and routine surveillance of antimicrobial resistance (AMR) to inform policy on the prevention and control of MDR Typhoid disease.

## Introduction

*Salmonella enterica* serovar Typhi (*S*.Typhi) is a Gram-negative bacillus in the Enterobacteriaceae family and is predominantly a human pathogen [1]. The pathogen is responsible for causing typhoid fever a systemic illness characterized by prolonged fever, headache, dry cough, and alteration of bowel habits [2]. Other common symptoms of the illness include fatigue, high fever (˃ 39°C), vomiting, coughing, and rapid pulse [3]. This disease poses a significant global health threat, with 5.6 billion people worldwide at risk of infection [4].

Living in densely populated areas, with poor sanitation and lack of safe drinking water, predisposes people living within urban slums in Africa and Asia to typhoid fever [5]. An incidence of 520 per 100,000 person-years of observation was found in children 8 years and below in Kenya’s largest slum, Kibera [6]. Mukuru settlement is one of the largest and most overcrowded urban slums in Nairobi, Kenya, with a population of approximately 250,000 [7]. As an informal settlement, it is characterized by poor infrastructure, sanitation, and a lack of safe water [8]. In addition, the patients can easily access antimicrobials over the counter perpetuating the emergence and spread of antimicrobial resistance [9].

Even after successful treatment, approximately 1–6% of typhoid infections become carriers [10]. The long-term persistence of *S*.Typhi in carriers explains why typhoid fever remains endemic in regions of the world with poor-quality drinking water and limited sewage treatment. *S*.Typhi dissemination occurs through the ingestion of food or water contaminated with faeces from typhoid patients and human carriers [11]. Acute infections and asymptomatic carriage caused by *S*.Typhi can be treated using effective antibiotics such as fluoroquinolones, however, the selection and spread of a highly resistant *S*.Typhi clade prevents such measures in areas with limited resources and irrational use of antibiotics [4]. The rise and spread of Multi-Drug Resistant (MDR) *S*.Typhi, strains resistant to 3 classes of antimicrobials: ampicillin, chloramphenicol, and sulfamethoxazole-trimethoprim, is a major public health concern because it increases patients’ hospital stays, it is costly to treat and increases the chance of mortality [12]. Over the years, because of the steady rise in antimicrobial resistance, the majority of typhoid cases in Kenya have been MDR coupled with reduced susceptibility to fluoroquinolones. Fluoroquinolones are the recommended drugs for the treatment of MDR typhoid in Kenya but the indiscriminate use of fluoroquinolones has led to a rapid increase in strains that are resistant to the antimicrobial. To prevent clinical treatment failure, it is critical to know the current trend of antimicrobial resistance in an endemic setting of typhoid fever like the Mukuru settlement.

For instance, Kavai *et al*., (2018) reported a Multi-Drug Resistance (MDR) prevalence of 55.5% of *S*.Typhi isolated from outpatient clinical samples from Mukuru villages in Nairobi [9]. Regular monitoring of the emergence of MDR *S*.Typhi and the dissemination of AMR-associated genes is crucial to gather essential information for making informed decisions on antibiotic usage [13]. It is therefore essential to investigate the typhoid carriage and circulating MDR strains in Mukuru. Our study aimed to determine the isolation rate of *S*.Typhi from patients and asymptomatic individuals and the MDR prevalence in Mukuru Informal settlement.

## Materials and methods

### Study design and site

A cross-sectional study design was used to determine the isolation rate of *S*.Typhi among cases and asymptomatic individuals in the Mukuru settlement and to identify and characterize antibiotic resistance genes within the same population. The study participants were recruited from three clinics in Mukuru informal settlement: Medical Missionaries of Mary (MMM), Municipal County Council Clinic (MCC), Mukuru Kwa Reuben Clinic (MR), and one inpatient facility, Mama Lucy Kibaki Hospital.

### Recruitment and laboratory procedures

#### Recruitment

Study participants were recruited from 12^th^ July 2021 to 5^th^ July 2022. These were outpatients who visited the four health facilities and presented with typhoid-like symptoms such as headache, diarrhoea/constipation, fatigue, and a temperature ˃ 37.5°C. After evaluation of the patient’s symptoms and inclusion criteria, the attending clinicians filled out the patients’ case report forms. The patients were then introduced to the study and made to understand its objectives by the research team. Patients who were willing to participate were taken through the informed consent or assent forms in their preferred language i.e., English or Kiswahili, and those who gave their consent were given a unique study identifier that was filled in on questionnaires and the same ID used on sample collection tubes. For every confirmed *S*.Typhi case, at least one contact of the patient living in the same household was requested to give a stool sample to check for carriage. Before sample collection, the contact was taken through the same ethical considerations described above and if found to be a typhoid carrier, the individual was treated.

#### Sample collection

The field research team guided the participants on how to use stool cups or rectal swabs. For children, their guardians were provided with rectal swabs and instructed on how to use them correctly. Rectal swabs were collected and dipped into Cary Blair media (Oxoid, Basingstoke, UK). Aseptically, blood samples were drawn from the arm by venipuncture and transferred directly into the blood culture bottle by qualified phlebotomists. 1-3ml and 8-10ml of blood were collected for children and adults respectively. Following the collection of the samples, a unique study identification number/barcode was labeled on the sample. Blood was transported at room temperature while stool/rectal swabs were transported at 4–8°C in cooler boxes to the CMR-KEMRI laboratory within 6 hours of collection.

#### Isolation and identification

Upon being received in the laboratory, rectal swabs were placed in selenite faecal enrichment broth and incubated at 37 ˚C overnight. From the enriched selenite F broth, streaking was done onto Xylose Lysine Deoxycholate agar (XLD) agar and Mac Conkey agar (Oxoid). Incubation was done at 37˚C for 24 hours. *S*.Typhi isolates were initially identified using distinguishing colony morphology characteristics such as pale-yellow colonies on MAC and brick red with black centres on XLD. Non-duplicate colonies from standard biochemical tests such as citrate, indole, urease, and Triple Sugar Iron (TSI) were examined with API20E (Biomeriux) and confirmed with serology. Serotyping tests were done using the slide agglutination technique using polyvalent antisera O- 9, and monovalent anti-sera d (Murex Diagnostics, Dartford, UK) [9].

Blood culture bottles were incubated in a BACTEC 9050 Culture System (Becton Dickinson, USA) at 37°C for up to seven days. A positive culture bottle with the reference strain was used to validate the results. Samples flagged as negative by the BACTEC were discarded while those flagged as positive were cultured onto MacConkey agar, Blood agar, and Chocolate agar. Isolates showing growth characteristics of *S*.Typhi were subcultured onto Mueller Hinton (Oxoid, Basingstoke, UK) for the growth of single discrete colonies.

#### Antibiotic susceptibility of isolates

The Kirby-Bauer disc diffusion technique was used in testing the antimicrobial susceptibility of *S*.Typhi isolates on Mueller-Hinton agar (Oxoid). The inoculum turbidity of the isolates was compared against the McFarland 0.5 turbidity standard. *S*.Typhi isolates were inoculated evenly on Mueller-Hinton Agar plates using the spread plate technique. The plates were then impregnated with antimicrobial sensitivity discs using sterile forceps and then gently pressed down onto the agar. Plates were then incubated at 37°C for 18 hours.

The following Oxoid^TM^ antibiotic disks were used; amoxicillin-clavulanate (AMC 20:10 ųg), nalidixic acid (NA 30ųg), ciprofloxacin (CIP 5ųg), ampicillin (AMP 10ųg), ceftazidime (CAZ 30ųg), cefotaxime (CTX 30ųg), ceftriaxone (CRO 30ųg), sulfamethoxazole-trimethoprim (SXT 1.25/23.57ųg), chloramphenicol (CHL 30ųg), and tetracycline (TET 30ųg)., cefpodoxime (CPD 10ųg) kanamycin (K 30ųg) azithromycin (AZT 15ųg).

For each *S*.Typhi isolate, two plates with antibiotics were used, labeled as Plate A and B. Plate A targeted Extended Spectrum Beta Lactamases (ESBL) production. The arrangement of antibiotics was as follows, penicillins (AMP), 3rd generation cephalosporin (CRO, CAZ, CTX, CPD), inhibitor at the middle (AMC). In plate B CIP, NA (targeting fluoroquinolones resistance), GEN, KAN, (targeting aminoglycosides resistances), CHL, SXT, and TET were also used because they are the recommended first-line drugs for the treatment of typhoid. *Escherichia coli* ATCC 25922 and *Staphylococcus aureus* ATCC 25923 were used for quality control of media quality and disc potency. Clinical and Laboratory Standard Institute (CLSI) guidelines were used to interpret zones of inhibition into susceptible, intermediate, and resistant. The isolates exhibiting resistance to chloramphenicol, ampicillin, and sulfamethoxazole-trimethoprim were classified as MDR [14].

#### Polymerase chain reaction

Out of the 54 *S*.Typhi isolates, twenty MDR isolates that showed resistance to ampicillin, chloramphenicol, and sulfamethoxazole-trimethoprim were further examined for the corresponding resistance genes using the conventional polymerase chain reaction. DNA was extracted using the boiling method. Briefly, a pea-sized amount of freshly cultured isolates was placed in 500μl PCR water (Invitrogen) in an Eppendorf tube. The mixture was then heated in a heating block at 95°C for 12 minutes, followed by centrifugation at 14000rpm for 5 minutes to obtain the supernatant. 200 μl of the supernatant was used as the template. The following resistance genes were tested; beta-lactamase genes (*bla*_*CTX-M*_, *bla*_*TEM-1B*_, *bla*_*SHV*_*) qnrS*, and *qnrA* for plasmid-mediated quinolones resistance. The Qiagen PCR kit (Qiagen) was used for amplification. The reaction mixture for PCR consisted of 12μl PCR water, 12 μl master mix (DNA polymerase, dNTPs, MgCL_2_, and buffer), 1μl of forward and 1μl reverse primer, and 1μl DNA template. PCR water was used as a negative control. Amplification was performed using published primers as summarised in **Table 1** in 0.2ml PCR tubes on a thermocycler.

**Table 1:** Shows primer sequences for ESBLs and quinolone resistance used in this study.

Target gene	Primer name	Primer sequence	Annealing T°C	Expected Bps	Reference
*bla*_*CTX-M*_	CTX-M-F	5′-ATGTGCAGYACCAGTAARGTKATGGC-3′	60°C	593	[15]
	CTX-M-R	5′-TGGGTRAARTARGTSACCAGAAYCAGCGG-3′			
*bla*_*TEM-1B*_	TEM-F	5’-TCGGGGAAATGTGCGCG-3’	50°C	851	[15]
	TEM-R	5’-TCGGGGAAATGTGCGCG-3’			
*bla*_*SHV*_	SHV-F	5′- TTCGCCTGTGTATTATCTCCCTG- 3′	50°C	854	[15]
	SHV-R	5′- TTAGCGTTGCCAGTGYTCG- 3′			
*qnrA*	QNRA-F	5’-AGAGGATTTCTCACGCCAGG-3’	55°C	580	[16]
	QNRA-R	5’-TGCCAGGCACAGATCTTGAC-3’			
*qnrS*	QNRS-F	5’-GCAAGTTCATTGAACAGGGT-3’	54°C	428	[16]
	QNRS-R	5’-TCTAAACCGTCGAGTTCGGCG-3’			

Amplification conditions were as follows, initial denaturation at 95°C for 5 minutes, 95°C for 30 seconds variable annealing temperature for 30 seconds, 72°C for 2 minutes 30 seconds, and a final extension at 72°C for 7 minutes. Annealing temperatures differed for the different genes as shown in **Table 1.**

### Ethical consideration

Ethical approval for the study was obtained from the Scientific Ethical Review Committee (SERU) of Kenya Medical Research Institute (No. KEMRI/SERU/CMR/P00156/4198). Written informed consent was obtained from adults participating in this study. For children, written informed consent was obtained from the parents/guardians, and verbal assent for older children 13–17 years was recorded. Unique identifiers in place of names were used to ensure that the participants’ anonymity was upheld. All the gadgets where data was stored were password protected and access to the data was strictly given to authorized staff.

### Data analysis

Data obtained in this study was entered into MS Excel. The isolation rate was determined by dividing typhoid-positive cases by the total number of study participants and expressed as a percentage. Frequencies and proportions of categorical data were analyzed using SPSS version 26. The zones of inhibition by disc diffusion were measured in millimeters and interpretation was done according to Clinical and Laboratory Standard Institute (CLSI) guidelines. WHONET was used to generate the graph depicting the distribution of resistance using disk diffusion zone diameters.

## Results

### Social demographics characteristics and isolation rate of typhoid

A total of 1014 participants with typhoid-like symptoms were recruited within one year from 2021 from four study sites. Of the 1014, the highest number of participants at 415 (41%) were from Medical Missionaries of Mary (MMM). In total, 516 (51%) were females while 498 (49%) were males. The overall *S*.Typhi isolation rate from the participants was 5% (54) with women having a higher isolation rate at 54% (29) compared to men at 46% (25) as shown in **Table 2**. Three (0.2%) of the 54 isolates were from asymptomatic carriers. These were contacts of positive cases who were requested to give a stool sample to check for carriage. In addition, the highest isolation rate was from MMM at 57% (31). The average age of the typhoid cases was 19 years with the oldest being 45 years and the youngest 8 months. The age group with the highest isolation rate from these findings was 21–30 at 44% (24).

**Table 2:** Social demographic characteristics of study participants.

Social demographic characteristics		Typhoid status		
		Negative *n* = 960	Positive *n* = 54	Total = 1014
**Gender**	Female	487 (51%)	29 (54%)	516 (51%)
	Male	473 (49%)	25 (46%)	498 (49%)
**Age_group**	0–10	577 (60%)	19 (35%)	596 (58.7%)
	11–20	88 (9%)	3 (6%)	91 (8.9%)
	21–30	125 (13%)	24 (44%)	149 (23.5%)
	31–40	102 (11%)	5 (9%)	107 (10.5%)
	41–50	45 (4.7%)	3 (6%)	48 (4.7%)
	51–60	16 (1.7%)	0 (0%)	16 (1.4%)
	61–70	5 (0.5%)	0 (0%)	5 (0.4%)
	71–80	2 (0.2%)	0 (0%)	2 (0.2%)
**Specimen_type**	Blood	0 (0%)	16 (30%)	16
	Stool	0 (0%)	38 (70%)	38
**Recruitment_facility**	MCC	216 (23%)	10 (19%)	226 (22%)
	MLK	142 (15%)	11 (20%)	153 (15%)
	MMM	384 (40%)	31 (57%)	415 (41%)
	MR	218 (23%)	2 (4%)	220 (22%)
**Participant_type**	0ut-patient	907 (94%)	51 (94%)	958 (94.5%)
	Contact	51 (5.3%)	3 (6%)	54 (5.3%)
	Emergency room	1 (0.1%)	0 (0%)	1 (0.01%)
	In-patient	1 (0.1%)	0 (0%)	1 (0.01%)

### Antimicrobial susceptibility patterns

The overall prevalence for the MDR phenotype was 37% (20/54). In addition, resistance to chloramphenicol was 39% (21/54) and 46% (25/54) both for ampicillin and sulfamethoxazole-trimethoprim. Moreover, the most common resistance phenotype was nalidixic acid where more than half of the isolates were resistant at 52% (28/54). A high proportion of the isolates showed reduced susceptibility towards ciprofloxacin which is the drug of choice for treating typhoid fever at 43% (23/54). All the isolates were susceptible to the aminoglycoside gentamicin but resistance to kanamycin was observed at 2% (1/54) as shown in **Table 3.** Interestingly, all the *S*.Typhi isolates analyzed were fully susceptible to the 3^rd^ generation of cephalosporins used in this study i.e., ceftriaxone, cefotaxime, ceftazidime and cefpodoxime.

**Table 3:** Percentage of antimicrobial resistance among the 54 *S*.Typhi isolates.

Antimicrobial	Susceptible	Resistant
	Number (%)	Number (%)
Ampicilin	29(54)	25 (46)
Ceftadizime	54 (100)	0
Ceftriaxone	54 (100)	0
Cefpodoxime	54 (100)	0
Cefotaxime	54 (100)	0
Amoxicillin clavulanate	53 (98)	1 (2)
Sulfamethoxazole-trimethoprim	29 (54)	25 (46)
Ciprofloxacin	31 (57)	23 (43)
Nalidixic acid	26 (48)	28 (52)
Azithromycin	49 (91)	5 (9)
Gentamicin	54 (100)	0
Kanamycin	53 (98)	1 (2)
Tetracycline	52 (96)	2 (4)
Chloramphenicol	33 (61)	21 (39)

**Fig 1:**
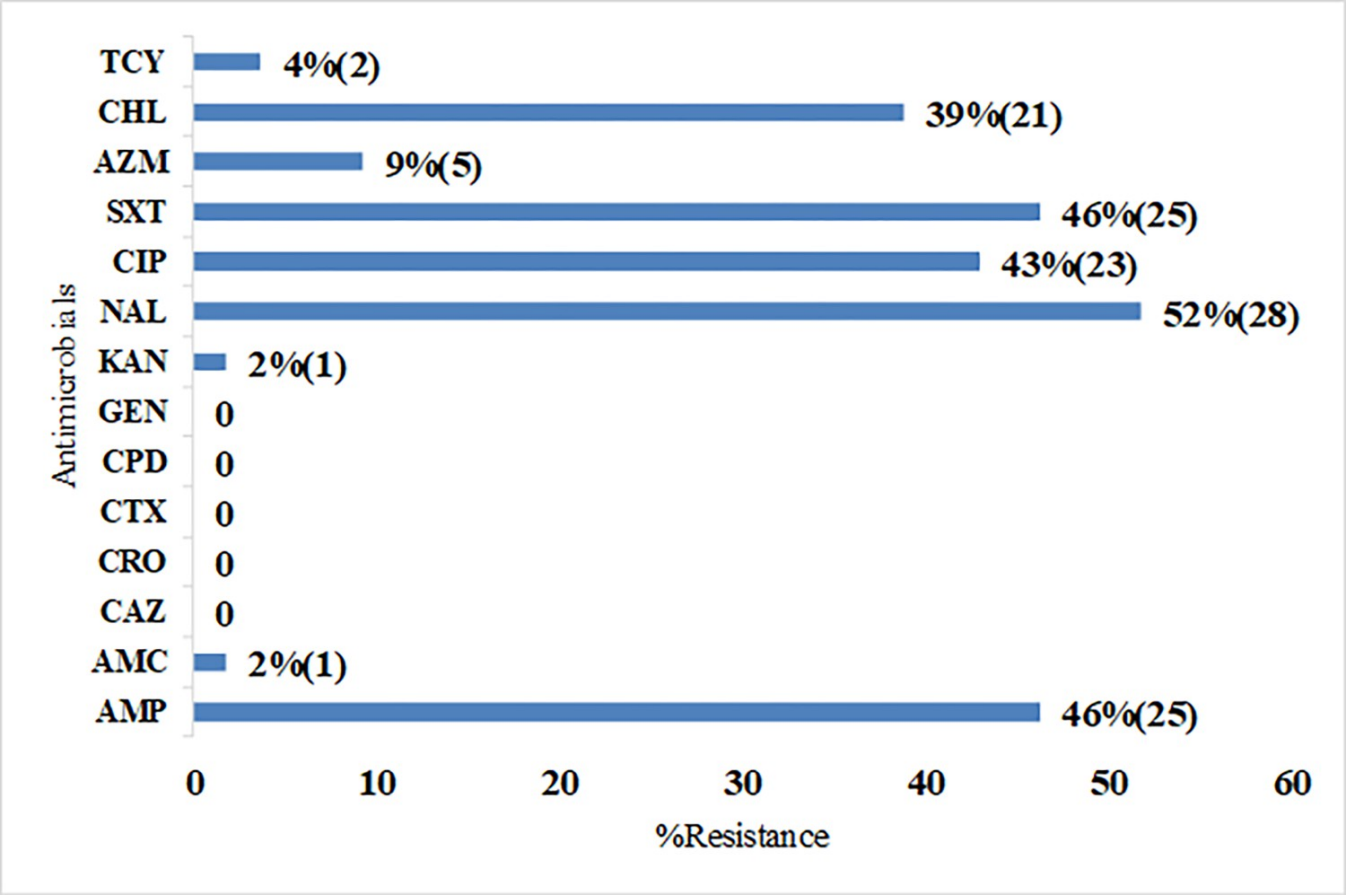
Antimicrobial resistance patterns (%) of isolated *S*.Typhi strains. KEY: Numbers in brackets represent the total number of resistant isolates for each drug. TCY-tetracycline, CHL-chloramphenicol, AZM-azithromycin, SXT- sulfamethoxazole-trimethoprim, CIP-ciprofloxacin, NAL-nalidixic acid, KAN-kanamycin, GEN-gentamicin, CPD-cefpodoxime, CTX-cefotaxime, CRO-ceftriaxone, CAZ-ceftazidime, AMC-amoxicillin clavulanate, AMP-ampicillin.

In addition, resistance to amoxicillin-clavulanate was recorded at 2% (1/54). Resistance to tetracycline and azithromycin was 4% and 9%, respectively as shown in **Fig 1**. Two out of three asymptomatic individuals harbored *S*.Typhi resistant to nalidixic acid and ciprofloxacin while one carrier harboured the *S*.Typhi MDR phenotype.

The resistance towards nalidixic acid was the highest across all the recruitment sites, ranging from 4 to 75%, as shown in **Table 4.** The resistance towards the first-line drugs; chloramphenicol, ampicillin, and sulfamethoxazole-trimethoprim was high in Medical Missionaries of Mary ranging from 50% to 62%, and was lowest in Mama Lucy Kibaki hospital ranging from 1–2% at 62%. The percentage resistance to ciprofloxacin which is currently the recommended drug for the treatment of typhoid in Kenya was highest in Medical Missionaries of Mary at 73% and lowest in Mukuru Kwa Reuben and Mama Lucy Kibaki Hospital at 4%.

**Table 4:** Antimicrobial resistance according to sampling site.

Study site	AMP	CAZ	CTX	CPD	CRO	SXT	AMC	CIP	NA	AZM	CN	K	TCY	CHL
Mukuru Kwa Reuben	2 (8%)	0 (0%)	0 (0%)	0 (0%)	0 (0%)	2 (8%)	0 (0%)	1 (4%)	1 (4%)	0 (0%)	0 (0%)	0 (0%)	0 (0%)	2 (10%)
Medical Missionaries of Mary	14 (56%)	0 (0%)	0 (0%)	0 (0%)	0 (0%)	15 (60%)	0 (0%)	17 (73%)	21 (75%)	2 (40%)	0 (0%)	0 (0%)	1 (50%)	13 (62%)
Mama Lucy Kibaki Hospital	2 (8%)	0 (0%)	0 (0%)	0 (0%)	0 (0%)	2 (8%)	0 (0%)	1 (4%)	1 (4%)	1 (20%)	0 (0%)	0 (0%)	1 (50%)	1 (5%)
Municipal City County Clinic	7 (28%)	0 (0%)	0 (0%)	0 (0%)	0 (0%)	6 (24%)	1 (100%)	4 (17%)	5 (19%)	2 (40%)	0 (0%)	0 (0%)	0 (0%)	5 (24%)

KEY: TCY-tetracycline, CHL-chloramphenicol, AZM-azithromycin, SXT- sulfamethoxazole-trimethoprim, CIP-ciprofloxacin, NAL-nalidixic acid, KAN-kanamycin, GEN-gentamicin, CPD-cefpodoxime, CTX-cefotaxime, CRO-ceftriaxone, CAZ-ceftazidime, AMC-amoxicillin clavulanate, AMP-ampicillin.

### PCR for selected resistance genes

**Fig 2:**
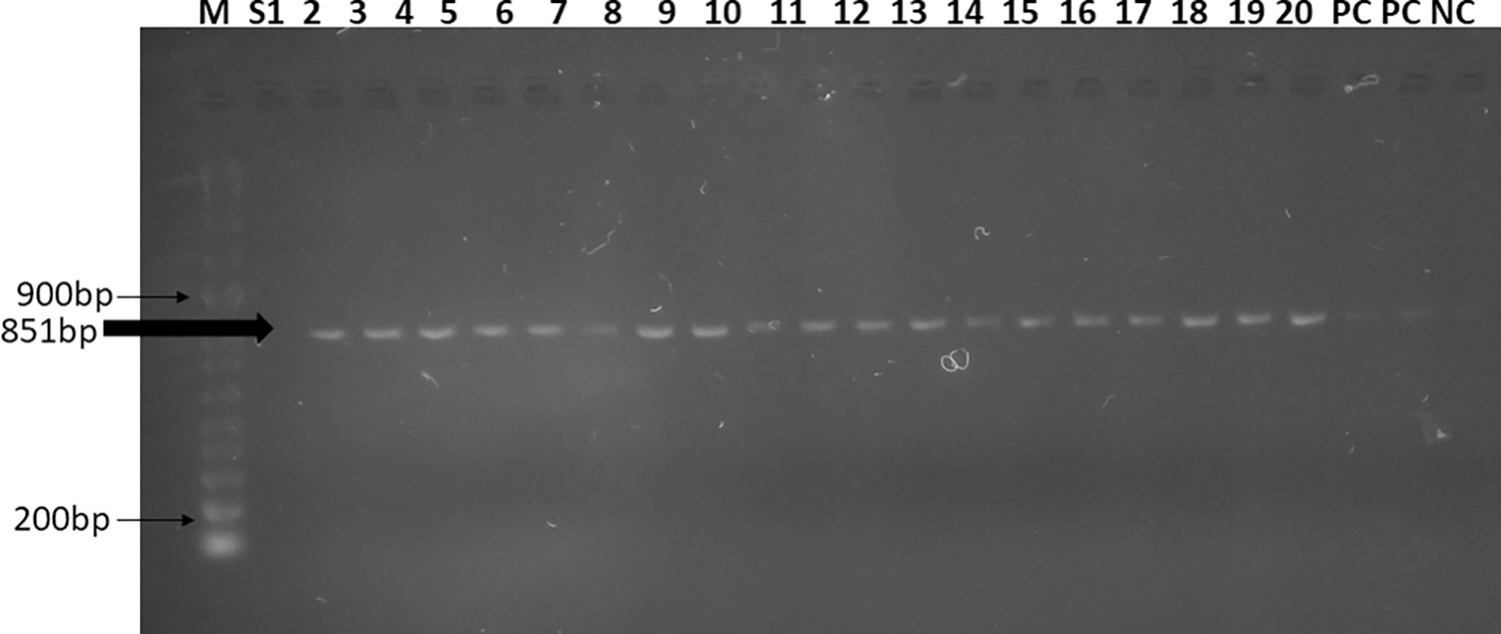
Gel electrophoresis of bla_TEM-1B_ positive MDR isolates. Abbreviations; M-molecular ladder (100-5000bp), NC-negative control, PC-positive control, S1-S20- MDR S.Typhi samples.

A total of twenty isolates exhibiting the MDR phenotype were screened for *bla*_*TEM-1B*_, *bla*_*CTX-M*_, *bla*_*SHV*_, *qnrB*, and *qnrS* genes. Of all the five genes, the beta-lactamase gene *bla*_*TEM-1*_ was positive and found present in 95% (19/20) of the MDR isolates, as shown in **Fig 2.**

## Discussion

In the current study, the overall isolation rate of *S*.Typhi was 5%, (54 *S*.Typhi isolated from 1014 patients). This isolation is higher when compared to another study by Kariuki *et al* (2021) which found an isolation rate of 3.2% [17]. This difference could be a result of the inclusion criteria where participants less than 16 years were sampled, but for this study, participants were recruited from 0–80 years. Of the 54 *S*.Typhi isolated in this study, three were from asymptomatic individuals giving an isolation rate of 0.2% from carriers in contrast to Kariuki *et al* (2019) who reported a carriage rate of 1% [9]. This variation in results could be attributed to the differences in the study participants’ inclusion criteria.

The isolation rate from stool was high at 70% (38/54) compared to 30% (16/54) from blood. A similar variation was observed by Kariuki *et al*. (2021) who reported an isolation rate of 58% from stool samples and 48% from blood [18]. The variation in the isolation rate between blood and stool specimens in this study could be attributed to the pathogenesis of the bacterium during the development of the disease. Ingested bacteria reach the small intestine where, through microfold (M) cells of Peyer’s patches, the bacteria migrate to the mesenteric lymph nodes, multiply, and are released into the bloodstream for the first time during infection[3,19]. *S*.Typhi disseminates causing transient primary bacteremia. Blood culture is the gold standard for detecting typhoid fever however its sensitivity decreases with the progression of the illness [20] In untreated cases, maximum blood culture yield is attained at the onset of typhoid fever during the first week of infection [19]. We assume that sample collection in our study could have been done when the participants were at the late stage of the disease.

In this current study, the highest isolation rate at 44% (24/54) was from the age group 21–30. In addition, it was also observed in the above-mentioned study that *S*.Typhi was more prevalent in patients over five years of age [17]. The difference in isolation rate is evidence that the prevalence of *S*.Typhi varies within populations over time.

The isolation of *S*.Typhi strains resistant to at least three different classes of antimicrobials; ampicillin, sulfamethoxazole-trimethoprim, and chloramphenicol, is evidence that the MDR strains still pose a health challenge in our community. MDR strains have been implicated in causing typhoid outbreaks [4]. We found an MDR prevalence of 37%. These findings are lower than Kavai *et al* 2018, who reported a prevalence of 55.5% in the Mukuru informal settlement [9]. The decrease could be because of the active vaccination strategies that have been deployed in such high-risk populations. Also, the decline could be attributed to different patterns of antimicrobial use or improved enactment of policies on prudent use of antimicrobials over the years during the implementation of the National Action Plan to combat and prevent Antimicrobial Resistance (AMR). In addition, our study findings are similar to Tack *et al*. 2019 who found an MDR prevalence of 38.3% in the Democratic Republic of Congo (DRC) [20].

Ciprofloxacin is the antimicrobial of choice for the treatment of typhoid fever caused by MDR *S*.Typhi [21]. Recommendation to use ciprofloxacin/ofloxacin over the years have resulted in the rise of resistance [20]. We recorded resistance to ciprofloxacin and nalidixic acid at 43% and 52%, respectively. These results are comparable to Mutai *et al*, *2019* who reported high resistance to ciprofloxacin at 69% in different settings. Typhoid infection by strains that are resistant to nalidixic acid has been linked to the causation of prolonged illness and hepatomegaly [22]. Various studies have highlighted a strong correlation between *S*.Typhi strains resistant to nalidixic acid/fluoroquinolones and poor clinical outcomes [23]. This implies that for successful treatment, other antimicrobial alternatives such as azithromycin and 3^rd^ generation cephalosporin should be considered.

For cases of complicated typhoid fever, third-generation cephalosporins are the drugs of choice [24]; our isolates were fully susceptible to this class of antimicrobials. However, these findings contrast those of Kavai *et al*, (2018) study, who reported less than 5% resistance towards third-generation cephalosporins [9]. Similarly, Tack *et al*. (2019) reported a 0.2% resistance towards 3^rd^ generation cephalosporins in a study done in DRC [20]. The variation in resistance levels could be attributed to them not being the first-line treatment for typhoid fever in the Mukuru settlement. Because no resistance was observed in our study phenotypically, the 3^rd^ generation cephalosporins, specifically ceftriaxone, can be effective in the treatment of typhoid fever in our endemic settings. Full susceptibility implies that *S*.Typhi isolates in this study did not harbour extended-spectrum beta-lactamase genes (ESBL) that could otherwise render the antimicrobial ineffective. This was confirmed by the absence of the ESBL *bla*_*CTX-M*_ gene by conventional PCR.

This study reports a high prevalence of the *bla*_*TEM-1B*_ gene at 95% (19/20 MDR isolates). These results are comparable with Kavai *et al* 2018, who reported an 80% prevalence of the *bla*_*TEM-1B*_ gene from *S*.Typhi isolated in selected Nairobi clinics [9]. Temporal variation when the studies were done could account for the differences in the prevalence. The *bla*_*TEM-1B*_ gene confers resistance to ampicillin and is associated with the emergence of ESBL-producing bacteria that are resistant to advanced cephalosporins [25]. The high prevalence of the gene implies that the strains are evolving to carry genes that make them survive in the presence of different classes of antimicrobials hence causing treatment failure that translates to therapeutic challenges with the available antibiotics for the treatment of typhoid.

*S*.Typhi resistance patterns are evolving at different rates with diverse phenotypic characteristics in various endemic areas [21]. Typhoid in the African region is known to be driven by the MDR *S*.Typhi. These strains harbor resistance genes in the incH1 plasmid such as *catA*, *sul1*, *sul2*, *dfrA*, *bla*_*TEM-1B*_, *strA*, *strB*, *tetA*, *tetB*, *tetC*, *and tetD* (4). These genes contribute to resistance to chloramphenicol, ampicillin sulfamethoxazole-trimethoprim, and tetracycline drugs [25].

It has been reported that chromosomal mutation in the *gyr*A gene is the most common source of reduced ciprofloxacin susceptibility in Africa [26]. Ciprofloxacin is designed to target DNA gyrase specifically GyrA, a protein that is essential in bacterial DNA replication [27]. Mutation on a single nucleotide at either codon position 83 or 87 of the gene (*gyrA*) encoding for the GyrA protein leads to resistance to nalidixic acid which translates to reduced susceptibility to fluoroquinolones like ciprofloxacin [4]. It has been discovered that resistance to nalidixic acid is a marker for fluoroquinolone resistance because mutations on the *gyrA* gene eventually evolve to cause resistance [27].

This study screened for the plasmid-mediated resistance genes; *qnrB* and *qnr*S. None of these genes were found present among the twenty MDR isolates. This shows that the resistance observed in our present isolates could be caused by a mutation in the chromosomally located *gyrA* gene. However, all the 54 isolates will be subjected to whole genome sequencing which will be able to provide comprehensive data of the resistance genes found in the *S*.Typhi strains.

Findings in this study show the current isolation rate of *S*.Typhi from an informal settlement including the trends in antimicrobial resistance across four sites. Comprehensive approaches to mitigate typhoid burden include improvements in hygiene and vaccination strategies which require identification of high-risk populations through continuous surveillance programs.

### Conclusion

MDR *S*.Typhi is prevalent in Mukuru Informal settlement posing a major public health threat to the population. The high resistance levels to nalidixic acid and reduced susceptibility to ciprofloxacin demonstrated in this study indicate that treatment of MDR *S*.Typhi by 3^rd^ generation cephalosporins will have better clinical outcomes. However, overreliance on antimicrobials is likely to cause the emergence of resistance. This reinforces the importance of ongoing surveillance to identify high-risk populations which will allow the implementation of public health interventions and research efforts to combat the MDR *S*.Typhi threat and typhoid fever in general. Because humans are the only source of infection and transmission of *S*.Typhi is by the fecal-oral route through contaminated water or food, prevention measures need to include provision of clean water and sanitation improvements, as well as health education.

### Study limitations

Determination of the true prevalence of typhoid carriers requires long-term follow-up of asymptomatic individuals. This study could not accomplish that because it utilized a cross-sectional study design which could have affected the accuracy of prevalence estimates. Thus, this design was not sufficient to understand typhoid fever trends in our settings. During the screening of resistance genes, only available primers at the time of analysis were used; hence other relevant resistance genes could not be observed. This limitation could be a potential area for future research to explore other resistance genes or conduct more comprehensive genomic analyses to better understand the genetic basis of resistance of the isolates.

## Supporting information

S1 File (XLSX)

S2 File (XLSX)
